# The contribution of personal and seniority variables to the presence of stress symptoms among Israeli UAV operators

**DOI:** 10.1186/s40696-016-0028-1

**Published:** 2016-11-29

**Authors:** Shiri Gal, Leah Shelef, Idit Oz, Nirit Yavnai, Erez Carmon, Shirley Gordon

**Affiliations:** 1 Psychology Branch, Israeli Air Force, Ramat Gan, Israel; 2 Mental Health Unit, Medical Corps, Israel Defense Forces, Ramat Gan, Israel; 3 IDF Medical Corps, Israel Defense Forces, Ramat Gan, Israel; 4 IAF, Ramat Gan, Israel

**Keywords:** UAV, Unmanned aerial vehicle, Anxiety, Depression, Post-traumatic stress disorder, PTSD

## Abstract

**Background:**

The exposure to war scenes via screens, despite offering a degree of detachment, can be stressful for the operator. The aim of the current study is to examine the existence of anxiety, depression, and post traumatic stress disorder (PTSD) symptoms among unmanned aerial vehicle (UAV) Israeli operators.

**Methods:**

Participants comprised 41 UAV operators (87.2% male), aged 22–38 (*M*_age_ = 26.05, *SD* = 3.54). Most (78.0%) reported having viewed battlefield scenes. All participants completed a total of five questionnaires: Beck Depression Inventory, State-Trait Anxiety Inventory, and three questionnaires of PTSD: Post Trauma Questionnaire (CAPS), the Post-Traumatic Cognition Inventory (CTPI), and the Post-Traumatic Symptom Scale (PSS).

**Results:**

Mean scores of depression and anxiety were found significantly lower than diagnosis cut-off points (*p* < .001). Senior operators showed higher means for depression (5.69 vs. 2.58, *p* = .040), of stress level (PSS; 3.17 vs. 0.25, *p* = .020) and for distress intensity (3.79 vs. 0.57, *p* = 0.041) than less-experienced operators.

**Conclusions:**

Investigating and monitoring the impact of battlefield exposure in UAV operators are highly beneficial for preventing psychopathology.

## Background

Over the past decade, due to technological developments, Unmanned Aerial Vehicles (UAVs) have become a major tool of war. The UAV is perceived as a machine—a robot that brought the battlefield into a sterile video-game-like environment. The UAV has become a critical asset, as it improves real-time intelligence, surveillance, and reconnaissance. The UAV provides close air support and increases precision strike operations on the battlefield [1].

Although UAV operators are not directly threatened during combat missions, as are infantry soldiers or fighter pilots, they are not immune to the trauma of war [2]. UAV operators are exposed to difficult sights, such as dead bodies, as if they were on the battlefield itself, occasionally even several times in the course of their military service [3]. Physically, UAV operators can be far from actual combat, but psychologically, they are situated on the battlefield. Their exposure via screens can be quite detached, yet stressful for the operators, as their actions may have a considerable impact on the course of the battle. This gap between the UAV operators’ physical and mental states may lead to reactions, such as helplessness and stress that, in turn, could affect their professional and interpersonal functioning [3].

Exposure to the battlefield and to life-threatening scenes, even if not being physically on the battlefield^3^, can be traumatic and may influence the development of further psychopathology, primarily posttraumatic stress disorder [4–8].

Research regarding psychological aspects associated with the operation of UAVs is limited. One study conducted on UAV operators who fought in Afghanistan showed that they were at greater risk for developing PTSD and showed significantly more symptoms related to combat stress, compared to soldiers from other units that had directly participated in battle [9]. A survey conducted among 296 UAV Predator/Reaper operators stationed within U.S. borders, supporting battlefield operations, revealed that 14–26% of the operators presented high levels of exhaustion and burnout [10]. A similar study, surveying 900 UAV operators fighting in Iraq and Afghanistan, found even higher numbers, reporting that 46% experienced high stress levels and 29% reported mental exhaustion and burnout [11]. The general assumption of these researchers was that the actual numbers are even higher, as not all UAV personnel were surveyed, the findings being limited to operators agreeing to participate in those studies [2].

Another factor placing UAV operators at risk for stress and depression symptoms, aside from their exposure to combat scenes, is their routine work schedule [1, 10]. UAV operators typically work in shifts, maintain long flight hours, and operate in an environment of manpower shortage. These time-based factors make it difficult to maintain normative family life.

Additional support to these findings can be found in a study of over 800 UAV operators [12]. This study highlighted an additional factor challenging the performance of the UAV operators: the need to maintain high levels of visual and auditory alertness for extended periods of time. This requirement had a great impact on the operators’ feeling of fatigue and burnout, a factor which could eventually lead to poor performance and in turn, increase feelings of helplessness and stress [13].

In light of the reviewed literature, queries arose regarding possible symptoms present among Israeli UAV operators, who are also exposed to complex scenes on a daily basis. The current study had two main objectives: first, to examine the presence of anxiety, depression, and PTSD symptoms; second, to examine the factors that may contribute to the development of these symptoms. Investigating and monitoring the impact of battlefield exposure in UAV operators could be highly valuable for preventing psychopathology and may facilitate the design of a prevention intervention by the Israeli Air Force (IAF) for this important population. To our knowledge, this is the first study of its kind conducted in the IAF and in the Israeli military.

## Methods

### Participants

The study population included 41 IAF UAV operators, some of whom were undergoing UAV training and some who were stationed in continuously active squadrons, aged 22–38 (*M*_age_ = 26.05, *SD*_age_ = 3.54), serving in one IAF base. 87.2% were male (*n* = 34), 80.5% (*n* = 33) were single. Over 70% of the participants (70.7%; *n* = 29) had more than 36 months seniority in their UAV operator job; 78.0% (*n* = 32) reported having had wartime experience, with the remaining 22.0% (*n* = 9) reporting experiencing on-going combat situations. Population characteristics are presented in Table 1.

**Table 1:** Study population (*N* = 41)

	n	%
Gender		
Male	34	83.0
Female	5	12.2
Missing data	2	4.8
Age		
≤25 years	18	53.0
>25 years	16	47.0
Family status		
Single	33	80.5
Married	8	19.5
Army seniority		
≤36 months	12	29.3
>36 months	29	70.7
Actual combat experience		
No	9	22.0
Yes	32	78.0

### Comparison variables

Since the sample included only UAV operators, we compared the independent variables—PTSD, depression, and anxiety within the group—by differences in demographic variables. For each demographic variable we created two sub-groups: gender (male/female); age (under/over age 25); family status (single/married); seniority (under/over 36 months, the compulsory service obligation); and experience in operational fighting (yes/no).

### Materials and procedure

Data collection was carried out from 14 April to 14 December 2014, after having received all necessary approvals from the IDF Human Research Review Board. The questionnaires were offered to all operators in all UAV squadrons.

The research tools comprised six self-administered questionnaires:

*Beck Depression Inventory*-*BDI* [14]. This questionnaire is comprised of 21 items which express affective, cognitive, somatic, and behavioral aspects of depression. Each symptom category describes different levels of depression, on a 4-point scale of 0-3, where 3 represents a high level of depression (e.g., *Lately:* 0. *I do not feel sad*; 1. *I am sad*; 2. *I am always sad, and I can’t get over* it; 3. *I am so sad or miserable that I can’t bear it*). Scores yielded for the entire questionnaire ranged from 0 to 63, where scores of 0–8 indicate a normal mood with no depressive symptoms; scores of 9–21 indicate depressive symptoms at a low level; scores of 22–29 indicate depressive symptoms; and scores of 30–63 indicate severe depression [14].

*State*-*Trait Anxiety Inventory*-*STAI* [15]. This 40-item questionnaire has two subscales: S-Anxiety (state anxiety refers to individuals’ level of anxiety in their current situation), having 20 items, and T-Anxiety (trait anxiety refers to individuals’ level of anxiety as a stable personality characteristic), having 20 items. In this study, we examined only state anxiety. The state-anxiety subscale asked respondents how they felt *right now*, using items tapping subjective feelings of apprehension, tension, nervousness, worry, and activation/arousal of the autonomic nervous system [16].

The S-Anxiety subscale is situated on a 4-point Likert-type scale, ranging from 1 (*does not describe me at all*) to 4 (*describes me a lot*). Sample state-anxiety items include *I am tense*; *I am worried*; *I feel calm*; *I feel secure*. Higher scores have been shown to be positively correlated with higher levels of anxiety [15]. Scores for the subscale ranged from 20 to 80, with higher scores indicating greater anxiety. A cut-off point of 39–40 has been suggested to detect clinically significant symptoms for the S-Anxiety scale [16].

*Post Trauma Questionnaire*-*CAPS* [17–19]. This 17-item questionnaire examines the frequency and intensity of traumatic symptoms, divided into three groups of PTSD symptoms: intrusion, arousal, and avoidance. Items such as the following characterized the questionnaire, presented on a 5-point Likert-type scale: *Have you ever had unwanted memories of (event)*? *Did they ever occur while you were awake or only in dreams*? Five response levels included *never* (0), *Once or twice* (1), *Once or twice a week* (2), *Several times a week* (3), *Daily or almost every day (4).* For determining a diagnosis on this questionnaire, it has been recommended to use the 1–2 rule: a frequency score of 1 (on the 5-point scale of 0–4) and an intensity score of 2 (on the 5-point scale of 0 = *none* to 4 = *extreme*) is required for a particular symptom to meet criterion [19]. The diagnosis is then made according to the DSM-IV algorithm (i.e., 1 *B* Criteria, 3 *C* Criteria, and 2 *D* Criteria, along with *A*, *E*, and *F*). A severity score for each symptom is calculated by summing the frequency and intensity scores, which can then be calculated for all 17 symptom questions or for the three symptom clusters, or for both (National Center for PTSD, Boston, USA) [20].

*The Post*-*Traumatic Cognition Inventory*-*PTCI* [21]. This 33-item questionnaire is used to measure anxiety, depression, and the presence of PTSD symptoms, by examining thoughts and beliefs related to trauma (negative cognitions about the self, about the world, and self-blame). The responses are rated on a 7-point Likert-type scale, ranging from 1 (*totally disagree*) to 7 (*totally agree*). Items such as the following appear in the inventory: *I have to be on guard at all times*; *I will never be able to feel normal emotions again*. Scale scores are formed for the three subscales, which show a high degree of intercorrelation (*r*s = .57–.75). Internal consistency appeared sound for the three subscales (Negative Cognitions about the Self, α = .97; Negative Cognitions about the World, α = .88; Self-Blame, α = .86) in the original sample [22].

*Post*-*Traumatic Symptom Scale*-*PSS* [22]. This questionnaire is a self-report instrument, comprising two subscales: Stress Level (PSS; 14 items) and Distress Intensity (PDS; 19 items), measuring the levels of stress the person experienced in the past two weeks. Items such as the following appear in the questionnaire, rated on a 4-point Likert-type scale, ranging from 0 (*not at all*) to 3 (*3*–*5 times per week or more/very much/very often*): *Experiencing physical reactions when reminded of the traumatic event (sweating, increased heart rate)*; *Feeling emotionally numb (unable to cry or have loving feelings*.

*Demographic questionnaire*. A questionnaire was designed for the purpose of the present study, comprising demographic questions such as gender, age, family status, seniority as a UAV operator, and professional combat experience in.

### Data analysis

Statistical analysis was conducted using SPSS (Version 21.0 for Windows). A significance level of *p* < .05 was adopted. One sample *t* test was employed to assess significance between known population threshold (cut-off) values and study population means. We used the value 8.00 as the cut off for depression (BDI) [14], and 38 for anxiety (STAI) [15]. Mean values of depression and anxiety were compared to these values, and were dichotomized by it for further analyses. An independent *t* test was conducted to assess differences between means of independent groups. For variables which did not present normal distribution, differences were assessed by using Mann–Whitney test.

## Results

Means of depression and anxiety were found significant when compared to the cut off values (4.78 ± 4.44, range 0–18, *p* < .001 and 33.05 ± 10.02, range 20–60, *p* < .001, respectively). Twenty-two percent of participants reported having mild depression (*n* = 9), according to the BDI score. A slightly higher number of participants (26.8%, *n* = 11) reported having anxiety, according the SATI score. Mean of post-traumatic symptoms for all participants for stress-PSS was 2.42 (*SD* = 4.49, range 0–20; *n* = 31), and distress intensity-PDS for all participants was 3.07 (*SD* = 5.23, range 0–20; *n* = 31). Extent and severity of traumatic symptoms-CAPS intensity was (*M* = 4.93, *SD* = 6.26, range 0–28; *n* = 28) and frequency (*M* = 5.18, *SD* = 6.41, range 0–28; *n* = 28) and negative thoughts-PTCI was (*M* = 55.44, *SD* = 20.10, range 16–93; *n* = 34).

The depression mean was doubled among operators whose seniority in their professional function was greater than 36 months, as compared to operators with fewer than 36 months of seniority (5.69 vs. 2.58, *p* = .040). Means, SD, statistics, and effect sizes are presented in Table 2.

**Table 2:** Depression and anxiety: means, SD, statistics, and effect sizes (*N* = 41)

Variables	Depression				Anxiety			
	n	M	SD	*p* value	n	M	SD	*p* value^1^
Total	41	4.78	4.44	–	41	33.05	10.02	–
Gender								
Male	34	4.65	4.44		34	33.00	9.04	
Female	5	5.40	4.83	0.728	5	33.40	16.41	0.935
Age								
≤25 years	18	3.94	3.80		18	32.67	9.32	
>25 years	16	6.38	4.75	0.107	16	36.19	10.33	0.304
Family status								
Single	33	4.42	3.94		33	32.09	9.08	
Married	8	6.25	6.21	0.302	8	37.0	13.20	0.218
Seniority								
≤36 months	12	2.58	3.03		12	29.75	8.69	
>36 months	29	5.69	4.65	0.040*	29	34.41	10.35	0.178
Actual combat experience								
No	9	3.33	2.40		9	31.33	6.54	
Yes	32	5.19	4.81	0.27 3	32	33.53	10.83	0.457

^1^Independent *t* test

It was also found that the means for stress level (PSS) and distress intensity (PDS) were significantly higher among operators whose seniority in their professional function was greater than 36 months, as compared to operators, whose seniority was less than 36 months (3.17 vs. 0.25, *p* = .020) and (3.79 vs. 0.57, *p* = .041), respectively (see Table 5).

In addition, the stress levels were found to be significant among operators older than 25, compared to younger operators (4.62 vs. 0.93, *p* = .030).

No significant differences were found in anxiety (see Table 2), extent and severity of traumatic symptoms (CAPS intensity and frequency; see Table 3), and negative thoughts (PTCI; see Table 4) between all independent variables. Means, SD, statistics, and effect sizes are presented in Tables 3, 4 and 5.

**Table 3:** CAPS frequency and CAPS intensity, means, SD, statistics, and effect Sizes (*N* = 28)

Variables	CAPS Frequency				s			
	n	M	SD	*p* value^1^	n	M	SD	*p* value^1^
Total	28	4.93	6.26	–	28	5.18	6.41	–
Gender								
Male	23	3.87	4.32		23	4.13	4.87	
Female	4	11.00	12.57	.202	4	10.75	12.04	.149
Age								
≤25 years	10	3.40	3.36		10	3.90	4.75	
>25 years	13	6.85	7.87	.303	13	6.85	7.96	.349
Family status								
Single	20	4.70	4.82		20	4.70	5.15	
Married	8	5.50	9.35	.758	8	6.38	9.16	.759
Seniority								
≤36 months	4	1.25	1.26		4	1.25	1.50	
>36 months	24	5.54	6.56	.196	24	5.83	6.69	.098
Actual combat experience								
No	6	3.67	3.00		6	3.83	2.86	
Yes	22	5.27	2.81	.713 s	22	5.55	7.08	.800

^1^Mann–Whitney test

**Table 4:** Negative thoughts (PTCI), means, SD, statistics, and effect sizes (*N* = 34)

Variables	Negative thoughts—total				Negative thoughts about the self				Negative thoughts about the world				Self-blaming			
	n	M	SD	*p* value^1^	n	M	SD	*p* value^1^	n	M	SD	*p* value^1^	n	M	SD	*p* value^1^
Total	34	55.44	20.10	–	34	28.296	9.45	–	34	18.21	9.91	–	34	8.94	5.58	–
Gender																
Male	28	53.93	18.90		28	27.21	8.60		28	17.64	9.38		28	9.07	5.99	
Female	4	60.75	27.86	.797	4	33.25	13.50	.818	4	18.75	12.58	.977	4	8.75	4.27	.660
Age																
≤25 years	15	50.33	14.13		15	25.47	4.17		15	16.33	7.84		15	8.53	5.87	
>25 years	15	60.87	24.61	.289	15	31.33	12.79	.203	15	20.87	12.07	.439	15	8.67	4.97	.543
Family status																
Single	26	55.04	20.41		26	28.0	9.88		26	18.12	9.89		26	8.92	6.06	
Married	8	56.75	20.36	.951	8	29.25	8.46	.790	8	18.50	10.65	.984	8	9.00	3.96	.509
Seniority																
≤36 months	8	51.25	14.79		8	26.38	5.37		8	18.38	9.62		8	6.50	1.41	
>36 months	26	56.73	21.56	.542	26	28.89	10.41	.683	26	18.15	10.18	.902	26	9.69	6.17	.135
Actual combat experience																
No	6	51.00	13.96		6	27.0	5.73		6	16.67	7.741		6	7.33	3.27	
Yes	28	56.39	21.27	.556	28	28.57	10.13	.820 s	28	18.54	10.41	.909	28	9.29	5.95	.378

^1^Mann–Whitney test

**Table 5:** Stress level (PSS) and intensity (PDS), means, SD, statistics, and effect sizes (*N* = 31)

Variables	Stress level—PSS				PDS intensity			
	n	M	SD	*p* value^1^	n	M	SD	*p* value^1^
Total	31	2.42	4.49	–	31	3.07	5.23	–
Gender								
Male	26	1.62	3.02		25	2.20	3.32	
Female	4	6.25	9.47	.477	4	5.50	9.71	.947
Age								
≤25 years	14	0.93	1.44		14	1.29	1.59	
>25 years	13	4.62	6.23	.030	13	5.69	7.25	.120
Family status								
Single	23	1.87	3.27		23	2.17	3.47	
Married	8	4.00	6.99	.635	8	5.63	8.33	.296
Seniority								
≤36 months	8	0.25	0.46		7	0.57	1.51	
>36 months	23	3.17	5.01	.020	24	3.79	5.72	.041
Actual combat experience								
No	6	1.17	1.17		6	1.67	1.86	
Yes	25	2.72	4.94	.958	25	3.40	5.74	.937

The contribution of personal and seniority variables to the presence of stress symptoms among Israeli UAV operators

^1^Mann–Whitney test

## Discussion

In general, results of this preliminary study did not indicate that UAV operators suffered from depression, anxiety, or PTSD symptoms. In other words, the differences found in the current study did not indicate that the participating UAV operators suffered from any clinical PTSD whatsoever. However, upon examining the personal and professional variables, a significant association was found between depression and seniority in the professional role. The mean of depression level among the senior operators was twice as high as that of the operators with less seniority (*p* = .04). Similarly, stress levels (PSS) and intensity of stress (PDS) were found to be significantly higher among operators with seniority of over 36 months, compared to those having seniority of fewer than 36 months (*p* = .020 and *p* = .041, respectively). In addition, stress levels were found to be significantly higher among operators above the age of 25, in comparison to those younger (*p* = .030).

These findings are in line with the Chappelle et al. [1] study of US army UAV operators, having found that, among the over-25-year-old participants, the more senior the operators, the greater their risk of developing PTSD symptoms. In Israel, the UAV operators’ work is structured so that the pressure grows in intensity with seniority. The current study did not examine the influence of long working hours and shift work on stress levels, a finding that had a considerable impact among US operators [1, 10]. This issue should be addressed in forthcoming studies, so as to extend the investigation of the human factor in operators following combat.

Moreover, no significant results were found in the anxiety index (STAI-T/S) [15], nor in two of the post-traumatic measures (CAPS, PTCI). These findings deviate substantially from the published literature for this population in the US Army. One explanation for the discrepancy could be the relatively small sample size in the current study. Another explanation could be the interaction of participants’ motivation with resilience factors. Since participating in the study was voluntary, only those volunteering completed the study’s questionnaires. It may be that the motivation to participate in the study is also related to personal resources, such as resilience, which characterizes those specific participants.

Even though the current survey was anonymous, the operators’ community is small, and some participants may have been concerned of being exposed. Furthermore, since both relatively elevated stress and depressive symptoms appeared in the more senior operators, this could be an outgrowth of cumulative career burnout rather than the current actual exposure to combat scenes. Further investigation most certainty needs to be carried out prior to drawing any conclusions.

A recent study conducted by Wood and his colleagues is noteworthy in the current context [23]. In this study, the authors assessed the prevalence of PTSD, using both objective measures and clinical interviews in remotely piloted aircraft (RPA). While no current cases of PTSD due to remote warfare were identified, they found higher levels of psychological distress and depressive and/or anxiety symptoms. These findings are in line with our findings. Most interestingly, Wood et al., found that those reporting higher levels of psychological distress did not identify their engagement in remote warfare as a significant contributing factor to these symptoms. The authors explained these findings as an outcome of strict screening processes, a procedure quite similar to that of the IDF.

In fact, to support the last idea, estimates in the aftermath of military service and/or participation in combat, of from 2 to 17% of veterans from various armies around the world, have been reported to suffer from PTSD [24, 25]. The rates of PTSD among Israeli veterans were 10–20% following the Yom Kippur War [26], 10–20% following the First Lebanon War [27], and 7–10% in the general civilian population during the second intifada (i.e., the Palestinian uprising that began in 2000) [28]. In summary, as can be seen, the percentage Israeli and US army veterans suffering from PTSD meeting full criteria falls within a similar range.

Still, regarding the generalizability of the current findings, it is important to consider that some parameters of the investigated population are unique. First, military service in Israel is mandatory for all citizens reaching the age of 18. Men serve three years in the IDF, while most women serve for only two years. UAV operators serve three years (36 months) in the framework of compulsory service, and continue to serve in reserve duty capacity, at least twice a month. So in effect, the UAV operators remain in continuous contact with their squadron, in addition to their civilian obligations. Israeli soldiers represent a mentally healthier population in relation to the general population [29], since, in advance of their enlistment, they undergo a series of screening tests and examinations in order to determine their suitability for military service [30].

Second, the selection procedure of UAV operators in the IDF is very arduous and complex. Eschewing details here, only a small portion of trainees actually complete the training, and are then required to serve five years (rather than the typical three years) of mandatory service, followed by a requirement for serving in reserve duty at least twice a month as mention above. This regimen may serve as a resilience factor for this population. Another resilience factor may be the prolonged exposure to missile attacks, parallel to those faced by the Israeli civilian population, so that the purpose and meaningfulness of their military action are bound up with the national reality, and thus can provide a strong sense of personal commitment and fulfillment.

## Conclusion

IDF UAV operators are not suffered from any clinical PTSD whatsoever, as found in previous study among US military UAV operators [9–11]. However, we can point of differences in psychological distress. Thus, investigating and monitoring the impact of battlefield exposure in UAV operators are highly valuable beneficial for preventing psychopathology.

But still, as noted, this study has several limitations. First, the study sample was relatively small. Second, a comparison group was not examined. Third, as in all studies based on self-report, it is difficult to evaluate the reliability of the participants’ responses and whether they reflect their authentic experience. Therefore, further investigation is in order.

In forthcoming studies, we would focus our efforts on a larger sample, examination and comparison of military population engaging in similar work experiences (being exposed to battlefield dynamics behind the screen, such as in military intelligence), to assess similarities and differences. In addition, we would focus on stressful situations, examining the level of stress or depression/anxiety symptoms regarding the type and nature of operational activity.

## Abbreviations

UAVs: unmanned aerial vehicles
PTSD: post-traumatic stress disorder
IAF: Israeli Air Force
IDF: Israel Defense Forces

## References

[1] Chappelle W, Goodman T, Reardon L, Thompson W (2014). An analysis of post-traumatic stress symptoms in United States Air Force drone operators. J Anxiety Disord.

[2] Miller G. Drone wars: are remotely piloted aircraft changing the nature of war? Science May 2012; vol 336. http://211.144.68.84:9998/91keshi/Public/File/41/336-6083/pdf/842.full.pdf.

[3] Zucchini D. Stress of combat reaches drones crew. Los Angeles Times 18 March 2012. http://articles.latimes.com/2012/mar/18/nation/la-na-drone-stress-20120318.

[4] Gates MA, Holowka DW, Vasterling JJ (2012). Posttraumatic stress disorder in veterans and military personnel: epidemiology, screening, and case recognition. Pscyhol Serv.

[5] Ikin J, Sim MR, Mckenzie DP (2007). Anxiety, post-traumatic stress disorder and depression in Korean War veterans 50 years after the war. Br J Psychiatry.

[6] Kullka RA, Schlenger WE, Fairbank JA (1990). Trauma and the Vietnam war generation: report of findings from the National Vietnam Veterans Readjustment Study.

[7] Solomon Z, Mikulincer M (2006). Trajectories of PTSD: a 20-year longitudinal study. Am J Psychiatry.

[8] Ramchand R, Schell TL, Karney BR, Osilla KC, Burns RM, Caldarone LB (2010). Disparate prevalence estimates of PTSD among service members who served in Iraq and Afghanistan: possible explanations. J Trauma Stress.

[9] Bumiller S. Air Force drone operators report high levels of stress, N.Y. Times 2011. The New York Times. http://www.nytimes.com/2011/12/19/world/asia/air-force-drone-operators-show-high-levels-of-stress.html. Accessed 26 Nov 2015.

[10] Ouma JA, Chappelle WL, Salinas A. Facets of occupational burnout among U.S. Air Force active duty and National Guard/reserve MQ-1 predator, MQ-9 reaper operators. Report no. AFRL-SA-WP-TR-2011-0003. OH: U. S. Air Force School of Aerospace Medicine: Wright-Patterson AFB, 2011.

[11] Chappelle W, McDonald K, Thompson B, Swearengen J. Prevalence of high emotional distress, symptoms of post-traumatic stress disorder in U. S. Air Force active duty remotely piloted aircraft operators (2010 USAFSAM survey results). Technical report AFRL-SA-WP-TR-2013-0002. OH: U. S. Air Force School of Aerospace Medicine: Wright-Patterson AFB, 2012.

[12] Chappelle WL, McDonald K, King RE. Psychological attributes critical to the performance of MQ-1 predator and MQ-9 reaper US Air Force sensor operators. No. AFRL-SA-BR-TR-2010-0007. Air Force Research Lab Brooks City-Base TX: Human Performance Wing (711th), 2010.

[13] Chappelle WL, McMillan KK, Novy P, McDonald K (2010). Psychological profile of USAF unmanned aerial systems Predator & Reaper pilots. Aviat Space Environ Med.

[14] Beck AT, Steer RA (1987). Manual for Revised Depression Inventory.

[15] Spielberger CD, Gorsuch RL, Lushene RE (1970). Manual for the State-Trait Anxiety Inventory.

[16] Julian LJ (2011). Measures of anxiety: State-Trait Anxiety Inventory (STAI), Beck Anxiety Inventory (BAI), and Hospital Anxiety and Depression Scale-Anxiety (HADS-A). Arthritis Care Res.

[17] Blake DD, Weathers FW, Nagy LM (1995). A clinician rating scale for assessing current and lifetime PTSD: the CAPS-1. J Trauma Stress.

[18] Weathers FW, Blake DD, Schnurr PP, et al. The clinician-administered PTSD scale for DSM-5 (CAPS-5). National Center for PTSD. http://www.ptsd.va.gov/professional/assessment/adult-int/caps.asp. Accessed Nov 26, 2015.

[19] Weathers FW, Ruscio AM, Keane TM (1999). Psychometric properties of nine scoring rules for the Clinician-Administered Posttraumatic Stress Disorder Scale. Psychol Assess.

[20] National Center for PTSD, Boston, USA. Clinician Administered PTSD Scale (CAPS). International Society for Traumatic Stress Studies. Retrieved Sept 24, 2015. http://www.istss.org/assessing-trauma/clinician-administered-ptsd-scale-%28caps%29.aspx.

[21] Foa EB, Ehlers A, Clark DM (1999). The posttraumatic cognitions inventory (PTCI): development and validation. Psychol Asses.

[22] Foa EB, Riggs DS, Dancu CV, Rothbaum BO (1993). Reliability and validity of a brief instrument for assessing post-traumatic stress disorder. J Trauma Stress.

[23] Wood J, Chappelle W, Correll T, Heaton J, Hubner M, McDonald K, Haynes JT. Prevalence of posttraumatic stress disorder in remotely piloted aircraft operators in the United States Air Force. May 2016.

[24] Creamer M, Wade D, Fletcher S, Forbes D (2011). PTSD among military personnel. Int Rev Psychiatry.

[25] Richardson LK, Frueh BC, Acierno R (2010). Prevalence estimates of combat-related post-traumatic stress disorder: critical review. Aust N Z J Psychiatry.

[26] Belenky GL, Noy S, Solomon Z, Del Jones F, Pichot P, Berner P, Wolf R, Thau K (1985). Psychiatric casualties (Battle Shock) in Israeli defense forces in the war in Lebanon June–September 1982. Psychiatry.

[27] Noy S, Nardi C, Solomon Z, Milgram NA (1986). Battle and military unit characteristics and the prevelence of psychiatric casualties. Stress and coping in time of war: generalizations from the Israeli experience.

[28] Hoffman YS, Diamond GM, Lipsitz JD (2011). The challenge of estimating PTSD prevalence in the context of ongoing trauma: the example of Israel during the Second Intifada. J Anxiety Disord.

[29] Gal R (1986). A portrait of the Israeli soldier.

[30] Bodner E, Ben-Artzi E, Kaplan Z (2006). Soldiers who kill themselves: the contribution of dispositional and situational factors. Arch Suicide Res.

